# *Chlamydia* Infection Across Host Species Boundaries Promotes Distinct Sets of Transcribed Anti-Apoptotic Factors

**DOI:** 10.3389/fcimb.2015.00096

**Published:** 2015-12-23

**Authors:** Joshua E. Messinger, Emmalin Nelton, Colleen Feeney, David C. Gondek

**Affiliations:** Department of Biology, Ithaca CollegeIthaca, NY, USA

**Keywords:** apoptosis, *Chlamydia*, evolution, emerging disease, transcription

## Abstract

*Chlamydiae*, obligate intracellular bacteria, cause significant human and veterinary associated diseases. Having emerged an estimated 700-million years ago, these bacteria have twice adapted to humans as a host species, causing sexually transmitted infection (*C. trachomatis*) and respiratory associated disease (*C. pneumoniae*). The principle mechanism of host cell defense against these intracellular bacteria is the induction of cell death via apoptosis. However, in the “arms race” of co-evolution, *Chlamydiae* have developed mechanisms to promote cell viability and inhibit cell death. Herein we examine the impact of *Chlamydiae* infection across multiple host species on transcription of anti-apoptotic genes. We found mostly distinct patterns of gene expression (Mcl1 and cIAPs) elicited by each pathogen-host pair indicating *Chlamydiae* infection across host species boundaries does not induce a universally shared host response. Understanding species specific host-pathogen interactions is paramount to deciphering how potential pathogens become emerging diseases.

## Introduction

Bacterial pathogens have co-evolved with their hosts, subverting host cell biology through secreted virulence factors to maintain a niche for pathogen growth. *Chlamydiae*, obligate intracellular gram-negative bacterial pathogens, have evolved from a common progenitor to become infectious specialists for a variety of host species (Nunes and Gomes, 2014). Two *Chlamydia* species, *C. trachomatis* and *C. pneumoniae*, predominate human infection. *C. trachomatis* infects epithelial cells of the reproductive tract, making it the number one bacterial sexually transmitted disease while *C. pneumoniae* can cause respiratory and in some cases atherosclerotic disorders (Campbell and Kuo, 2004; Starnbach and Roan, 2008). *Chlamydiae* replicate within an intracellular compartment called the inclusion. To maintain this replicative cellular niche, *Chlamydiae* have evolved mechanisms to prevent host cell death, one in particular being the inhibition of apoptosis.

Apoptosis is an organized mechanism of cellular death triggered through either an extrinsic pathway (death receptor signaling) or a cellular intrinsic pathway (mitochondrial breakdown). A common feature in both these pathways is the activation of cytosolic proteases, called caspases (Thornberry and Lazebnik, 1998). While the extrinsic pathway activates caspases directly, the intrinsic pathway requires destabilization of the mitochondrial membrane, mediated by pro-apoptotic proteins such as Bax, Bak, and Bad, leading to the release of cytochrome C. The pro-apoptotic proteins can be held in check by cellular anti-apoptotic factors such as Bcl-2, Mcl-1, and cIAP. Both the intrinsic and extrinsic apoptosis pathways of apoptosis result in nuclear fragmentation and organized dispersal of cellular contents via membrane blebbing.

Intracellular bacterial infection requires host cell survival. Previous studies have shown that *Chlamydia* infection results in the inhibition of the host cell apoptotic response under a variety of conditions (Fan et al., 1998). *Chlamydia* can inhibit the cleavage of pro-apoptotic proteins (Xiao et al., 2004). Infection can also cause the upregulation of host anti-apoptotic factors such as MCL1 and IAP complexes (Rajalingam et al., 2006, 2008). However, conflicting reports exist in the literature regarding the impact of these anti-apoptotic factors. For example, *Chlamydia trachomatis* infection of human cells requires cIAP complexes, whereas these complexes are not essential for infection of mouse cells by this same pathogen (Ying et al., 2008).

To address this discrepancy, we took an evolutionary medicine approach. The infectious process places significant genomic pressures on both the pathogen and the host in the “arms race” of co-evolution (Red Queen Hypothesis) (Van Valen, 1973). This hypothesis captures the idea that as the host evolves mechanisms to sense infection and eliminate the invader, the pathogen is coordinately evolving mechanisms to shut down those host processes. Since the radiation of *Chlamydiae* has occurred over the past few million years, it is likely that each pathogen species has evolved host specific adaptations to ensure survival (Borges et al., 2012). Cellular apoptosis is a highly conserved mechanism for combating intracellular infection and *Chlamydiae* have been shown to repress this response in cells upon infection. However, there is no evidence that all *Chlamydiae* use the same anti-apoptotic mechanism to reach that outcome.

Here we show that *Chlamydiae* infection directly impacts host transcriptional levels of anti-apoptotic proteins. The addition of cycloheximide (CHX), a eukaryotic translational inhibitor, significantly impacts this host transcriptional profile, indicating a potential positive feedback loop of anti-apoptotic gene activation elicited by the pathogen. Utilizing three separate *Chlamydiaceae* (*C. trachomatis*-Ct, *C. muridarum*-Cm, *C. caviae*-Cc) and multiple host cell lines (human, mouse, guinea pig, monkey, and cat) we identify that each pathogen induces a unique anti-apoptotic transcriptional response dependent upon host species infection. Examining the host pathogen interactions and comparing infections will allow us to understand the boundaries for cross species infectivity. This insight is key to unraveling the evolution of new and emerging disease.

## Methods

### Cell culture and *Chlamydiae* stocks

McCoy (mouse fibroblast) and 104C1 (guinea pig lung fibroblast) cells were maintained in RPMI media supplemented with 10% FBS (Atlanta Biologics), L-glutamine, HEPES buffer, and β-mercaptoethanol. Vero (African green monkey kidney) and HeLa (Human endocervical) cells were maintained in Dulbecco's Modified Eagles Medium (DMEM) supplemented with 10% FBS plus additional components listed above. AK-D (Cat lung fibroblast) cells were maintained in Hams-F12 media with 10% FBS plus additional components above. All cells were grown at 37°C with 5% CO_2_. *Chlamydia trachomatis* (L2/434Bu and D/UW-3/CX), *Chlamydia muridarum* (MoPN), and *Chalmydiophila caviae* (GPIC) bacterial stocks were purified over a renografin gradient, resuspended in sodium phosphate glutamate (SPG) and stored at −80°C. To titer stocks, purified *Chlamydiae* were serial diluted on McCoy cells and centrifuged for 1 h at 1500RCF at 37°C. Cells were incubated for 30 h in media containing cycloheximide (CHX) (1.5 μg/mL) prior to fixation and staining. *C. trachmoatis* and *C. muridarum* strains were generously provided by the Starnbach Lab (Harvard Med) and C. caviae was purchased from ATCC.

### Immunofluorescent staining

After overnight methanol fixation, cells were rehydrated and stained with primary antibody (αHsp60—Thermo A57B9), secondary antibody (AlexaFluor A11032) and Hoechst 33258 or DAPI. Immunofluorescent stains were imaged using a Nikon Eclipse E800 UV microscope with a Nikon DS-Ri1 camera adapter. Images were analyzed with Nikon NIS-Elements D imaging software. For quantification and unbiased analysis of infectous units per milliliter images were processed using MATLAB image processing toolbox (mathworks.com). Within MATLAB, a script was written to batch convert images to grayscale, uniformly threshold the images, and then count numbers of inclusions (dots) per image. Data were exported to Excel for further analysis and quantification of infectious units per milliliter of lysate.

### Recoverable infectious unit assay

Confluent monolayers of McCoy, 104C1, AK-D, Vero, and HeLa cells were plated on Costar tissue culture 24 well plates for overnight growth in the conditions mentioned above. Monolayers were infected at an MOI of approximately one (1 × 10^5^ IFU) by *C. trachomatis, C. caviae*, and *C. muridarum* in media by centrifugation at 37°C at 1500 RCF for 1 h. Media was changed and the *Chlamydia* were allowed to grow in the presence or absence of CHX (1.5 μg/ml) at 37°C. At 30 h post infection media was collected into a new Costar 24 well plate and cells were hypotonically lysed using sterile water. Lysates were vigorously pipetted prior to being combined with the spent media and the mixture was frozen at −80°C to ensure freeze fracture of infected cells. Cell lysates were serial diluted in SPG over confluent monolayers of McCoy cells in Costar 96-well plates and were centrifuged as described above.

### Cell death and apoptosis assay

Confluent monolayers of McCoy, HeLa, and 104C1 cells were plated on Costar 96-well tissue culture plates for overnight growth. Cells were infected with *C. trachomatis* at an MOI of approximately five, or *C. caviae* or *C. muridarum* at an MOI of approximately two in SPG using centrifugation as previously described (Coers et al., 2011). Following centrifugation, media was replaced with or without CHX (1.5 μg/ml) and allowed to incubate at 37°C for 30 h. At 36 h post infection, cells were challenged with staurosporine (104C1–25 μM, McCoy–10 μM, HeLa–2 μM) and allowed to incubate for 4 h at 37°C. Cells were then stained for immunofluorescence microscopy as described above.

### Gene expression analysis by RT-qPCR

Genomic sequences for the following genes were obtained through the Ensembl bioinformatic database (ensemble.org). All known isoforms were analyzed and the primer binding sites that were flanking exon boundaries were determined for shared exons in all isoforms. Upon identifying exon boundaries, these sequences were entered into PRIMER3 (http://bioinfo.ut.ee/primer3/) to determine thermodynamically ideal primers for RT-qPCR(see table below) of genes previously identified as modulated by Chlamydia infection (Wahl et al., 2003; Rajalingam et al., 2006, 2008; Kun et al., 2013). Confluent monolayers of McCoy, HeLa, and Guinea Pig cells were plated in Costar six well tissue culture treated plates for overnight growth. Cells were infected at an MOI of approximately five for *C. trachomatis* and an MOI of approximately two for *C. caviae* and *C. muridarum*. At 30 h post infection, media was aspirated, cells washed, and lysed directly in the plate for total RNA isolation as directed by the Omega Biotech Total RNA Isolation Kit. RNA was stored at −80°C before analysis through RT-qPCR. All 1-step RT-qPCR reactions (Quanta BioSciences) were generated as directed by the manufacturer and analyzed on a Roche454 Lightcycler.

Primer Sequences Used in this Study

**Table:** 

		**Forward 5′—3′**	**Reverse 5′—3′**	**Product**
Guinea pig	cIAP1	GCCATCTACGGTTCCAATTC	CACCATCACAGCAAAAGCAT	97
	cIAP2	Gene is not annotated in database		
	Bcl2	TCCCAGAGAGGCTACGAGTG	ACACCAAGCGGGGAGTTG	109
	Mcl1	CACCAGCAGGAAGGCACT	ATGTCCAGTTTCCGAAGCAT	96
	Survivin	GAACTGGCCCTTCGTGAAC	AAGAAACACTGCGCCAAATC	107
	XIAP	TTGGGACATGGACGTATTCA	TCCTCCTCCACAGTGAAAGC	103
	GAPDH	CCTAATGTGTCGGTTGTGGA	TGTTGAAGTCACAGGACACAA	150
Mouse	cIAP1	GTTGTGATGGTGGCTTGAGA	CTCCTGACCCTTCATCCGTA	106
	cIAP2	GGGGAGTTCCTGTGTCAGAA	TGTCTAGCATCAGGCCACAG	101
	Bcl2	AGTACCTGAACCGGCATCTG	CAGCCAGGAGAAATCAAACAG	109
	Mcl1	TGTAAGGACGAAACGGGACT	ATTTCTGATGCCGCCTTCTA	98
	Survivin	ATCGCCACCTTCAAGAACTG	AATCAGGCTCGTTCTCGGTA	105
	XIAP	TGGAACATGGACATCCTCAG	CCTCCACAGTGAAAGCACTTC	98
	GAPDH	CAATGTGTCCGTCGTGGA	GTTGAAGTCGCAGGAGACAAC	147
Human	cIAP1	TGCGGCCAACATCTTCAAAA	CGTTCTTCTTGCAACCTCCTC	150
	cIAP2	TGGGTTCAACATGCCAAGTG	TCATCTCCTGGGCTGTCTGA	140
	Bcl2	AGTACCTGAACCGGCATCTG	CAGCCAGGAGAAATCAAACAG	119
	Mcl1	CTTACGACGGGTTGGGGATG	GCGGAAACAATGACTCATGGC	150
	XIAP	TGGCAGATTATGAAGCACGGA	TAGCCCTCCTCCACAGTGAA	137
	GAPDH	AACGGGAAGCTTGTCATCAATGGAAA	GCATCAGCAGAGGGGGCAGAG	194

### Caspase-3 colorimetric absorbance assay

Cells were plated into six well plates (5^*^10^5^ per well) for overnight growth. Cells were infected as described above. Twenty-six hours post infection cells were left either untreated or treated with staurosporine for 4 h prior to collection. Cells were probed for caspase three activity using the commercially available Caspase-3 Cellular assay kit PLUS (Enzo Life Sciences). Absorbance readings at 405 nm were made every 10 min for 6 h using a TECAN NanoQuant infinite M200 Pro plate reader.

### Statistical analysis

All experiments were independently replicated two or more times as noted in figure legends. Statistical significance was determined via One-way ANOVA and Tukey's *post-hoc* pairwise comparison test. *P*-values less than 0.05 were used to determine significant data.

## Results

### *Chlamydiae* infection and growth across host species boundaries

A pathogen and its host are under constant genomic pressure as the duo are locked in coevolutionary arms race of adaptation (Red Queen Hypothesis; Van Valen, 1973). Upon infection, pathogen recognition receptors (NOD/TLR) are stimulated in the cell leading to changes in gene transcription and protein activation (O'Connell et al., 2006). These pathways can elicit the production of new proteins which result in pathogen elimination and/or cell apoptosis. *Chlamydiae* have adapted host-specific mechanisms to subvert this cellular response by modulating the host response to infection at a transcriptional and post-translational level (Hess et al., 2001; Olive et al., 2014).

**Figure 1:**
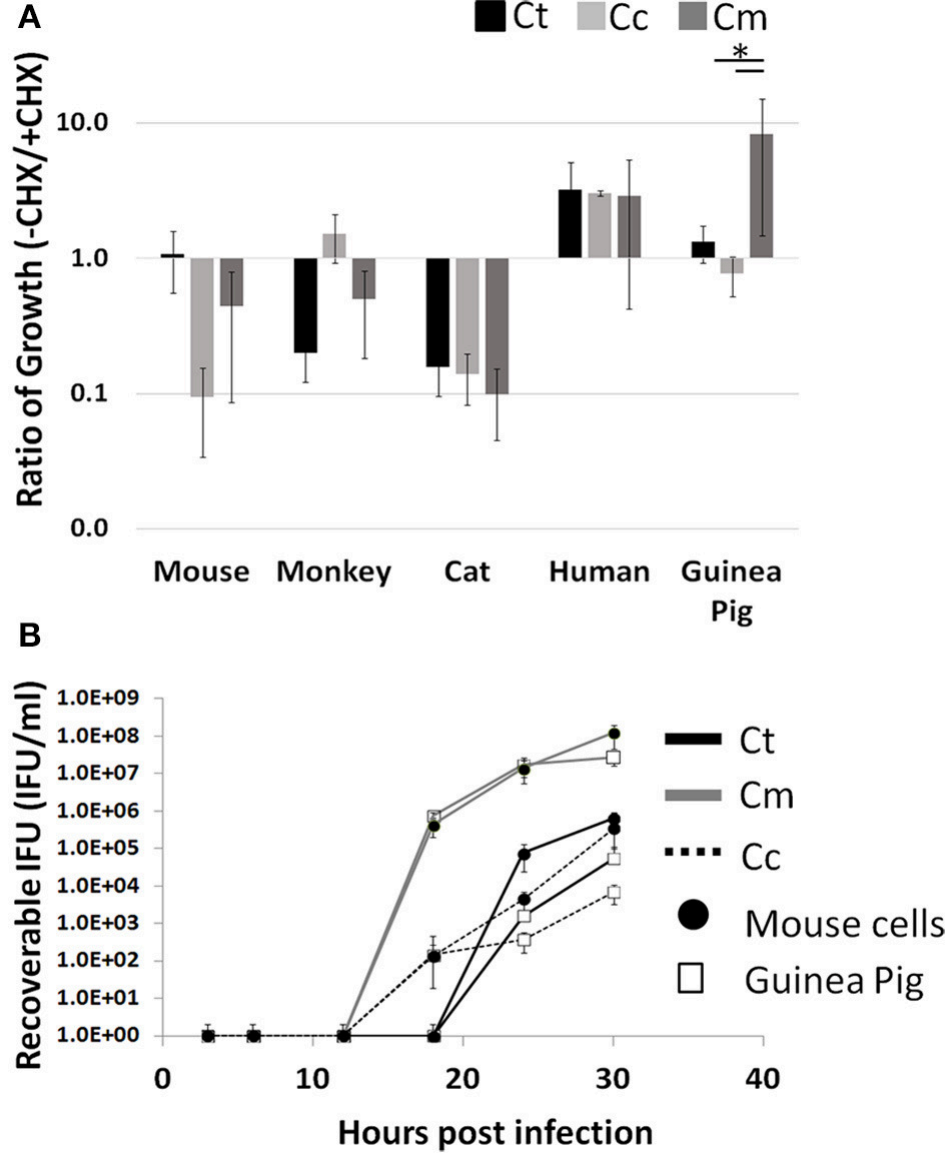
**Enhanced growth in the presence of host cell translation—Cells were infected in 24 well plates (10^**5**^ cells/well) for 30 h with or without CHX (1.5 μg/mL)**. Supernatants of hypotonically lysed cells were clarified by centrifugation and titered. *Chlamydiae* were stained with α-HSP60 to quantitate infectious units/ml **(A)**. Presented is the ratio of (-CHX/CHX). Growth curves of Cm and Ct (MOI = 1 at 1 × 10^5^ IFU) were determined in mouse and guinea pig cells (Gray line-Cm, solid line-Ct, dashed line-Cc open marker—guinea pig, solid marker—mouse) **(B)**. Ct-*C. trachomatis*, Cc-*C.caviae* Cm-*C.muridarum.* ^*^p < 0.05 (data are representative of two independent trials).

Cycloheximide (CHX) is a commonly used eukaryotic translation inhibitor that improves the growth of *Chlamydia* by reducing competition for intracellular amino acids (Allan and Pearce, 1983b). Thus, this treatment is able to improve the growth of poorly host adapted pathogens. A well-adapted pathogen would not need this “advantage.” Moreover, this CHX block of host cell translation could inhibit *Chlamydia's* ability to manipulate the infected host cell. We hypothesized that host adapted *Chlamydia* relies on active host cell translation to reach maximal growth potential. To test this, we examined the impact of CHX on the ability to enhance or inhibit pathogen growth across host species boundaries. We infected the following five host cell lines with each pathogen (Ct, Cc, and Cm): mouse, monkey, cat, human, and guinea pig. To compare growth potential, we took the ratio of total infectious progeny in the absence vs. presence of CHX such that a positive correlation indicates improved growth due to ongoing host translation. We found that only Cm in the guinea pig cell line was able to grow significantly better in the presence of host cell translation, compared to Ct and Cc (*p* < 0.05; Figure 1A).

To determine if this high infectious load in guinea pig cells is a result of a more rapid conversion to the infectious elementary body, we compared Cm and Ct growth in guinea pig to that in mouse cells (Figure 1B). The *Chlamydia* IFU production during Cm infection appeared to be more rapid than Ct or Cc infection. Cm loads were similar between guinea pig and mouse cell lines across all time points. For Ct and Cc, infections of the Guinea Pig cell line typically yielded one log lower IFU at 30 h post infection, compared to McCoy cell infection. Additionally, Cm demonstrated high bacterial loads in all host species cell lines tested compared to Ct and Cc infections (Supplemental Figure 1).

### Apoptosis inhibition is a key determinant of *Chlamydiae* growth potential

**Figure 2:**
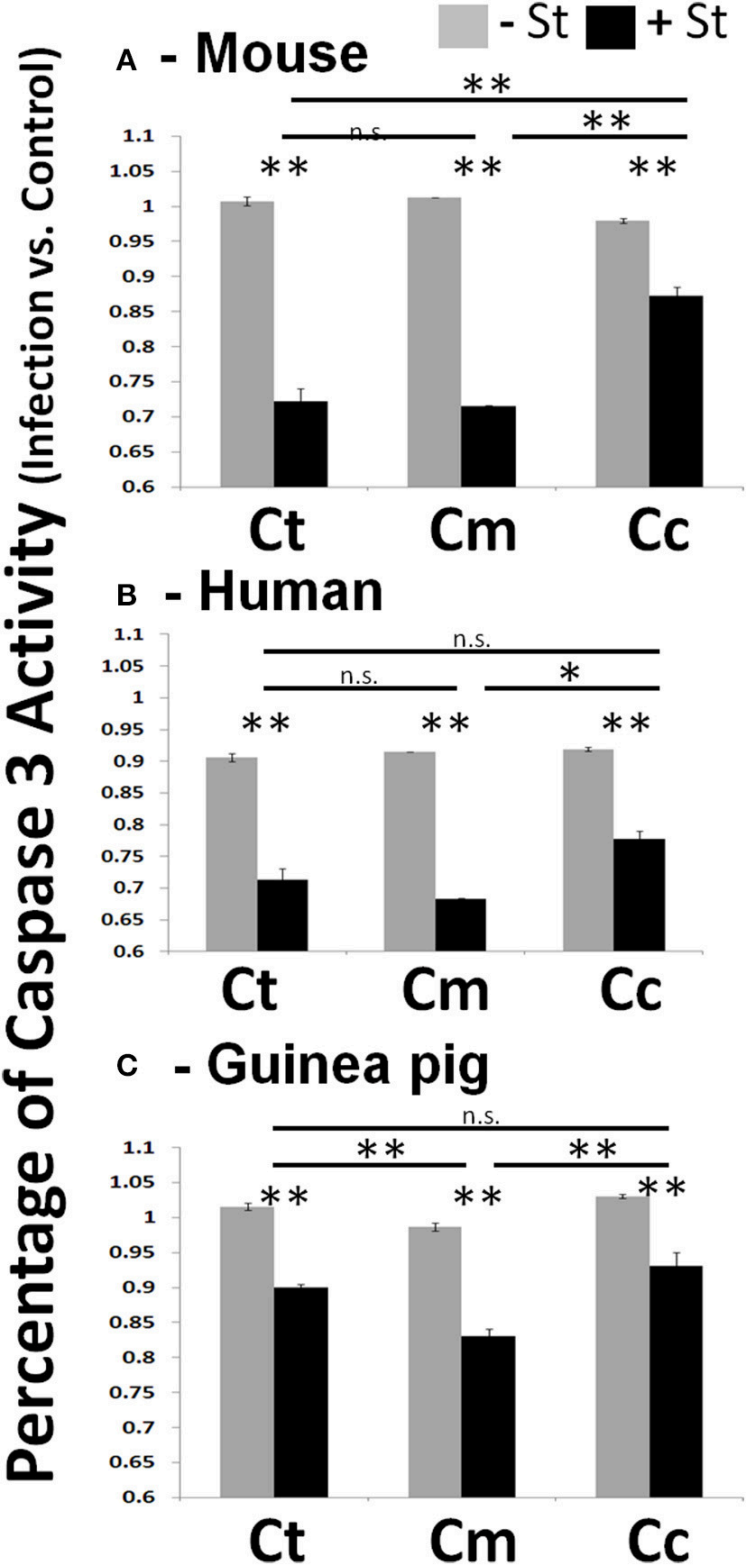
*****Chlamydiae*** inhibit Caspase three activation in diverse hosts—Cell lines were infected with the indicated pathogens and allowed to incubate for 26 h**. Staurosporine was added for 4 h before cell lysates were collected for colorimetric analysis of caspase three activity in mouse cells **(A)**, human cells **(B)**, and guinea pig cells **(C)**. Ct-*C. trachomatis*, Cc-*C.caviae* Cm-*C.muridarum*. ^**^p < 0.01, ^*^p < 0.05 (representative of greater than three independent trials).

In experiments where *Chlamydiae* growth potential was particularly poor, cells examined by microscopy displayed cellular blebbing and a significant debris field, indicative of apoptosis (Supplemental Figure 2C). As maintenance of host cell viability is absolutely critical for success of obligate intracellular bacteria, a host-adapted pathogen would be able to inhibit cellular apoptosis. To test this hypothesis, we focused our studies on mouse, human, and guinea pig host species. We infected each host cell species with *Chlamydiae* followed by treatment with staurosporine, to induce host cell apoptosis. We examined the production of active caspase three and fragmentation of nuclei in order to determine the extent of apoptosis inhibition. As shown in Figure 2, cells infected with each species of *Chlamydiae* were able to inhibit the activation of caspase three while uninfected cells could not. Cm exhibited the most pronounced inhibitory activity across host species boundaries, particularly in guinea pig cells, whereas Cc was the most limited in its ability to block caspase 3 activity.

**Figure 3:**
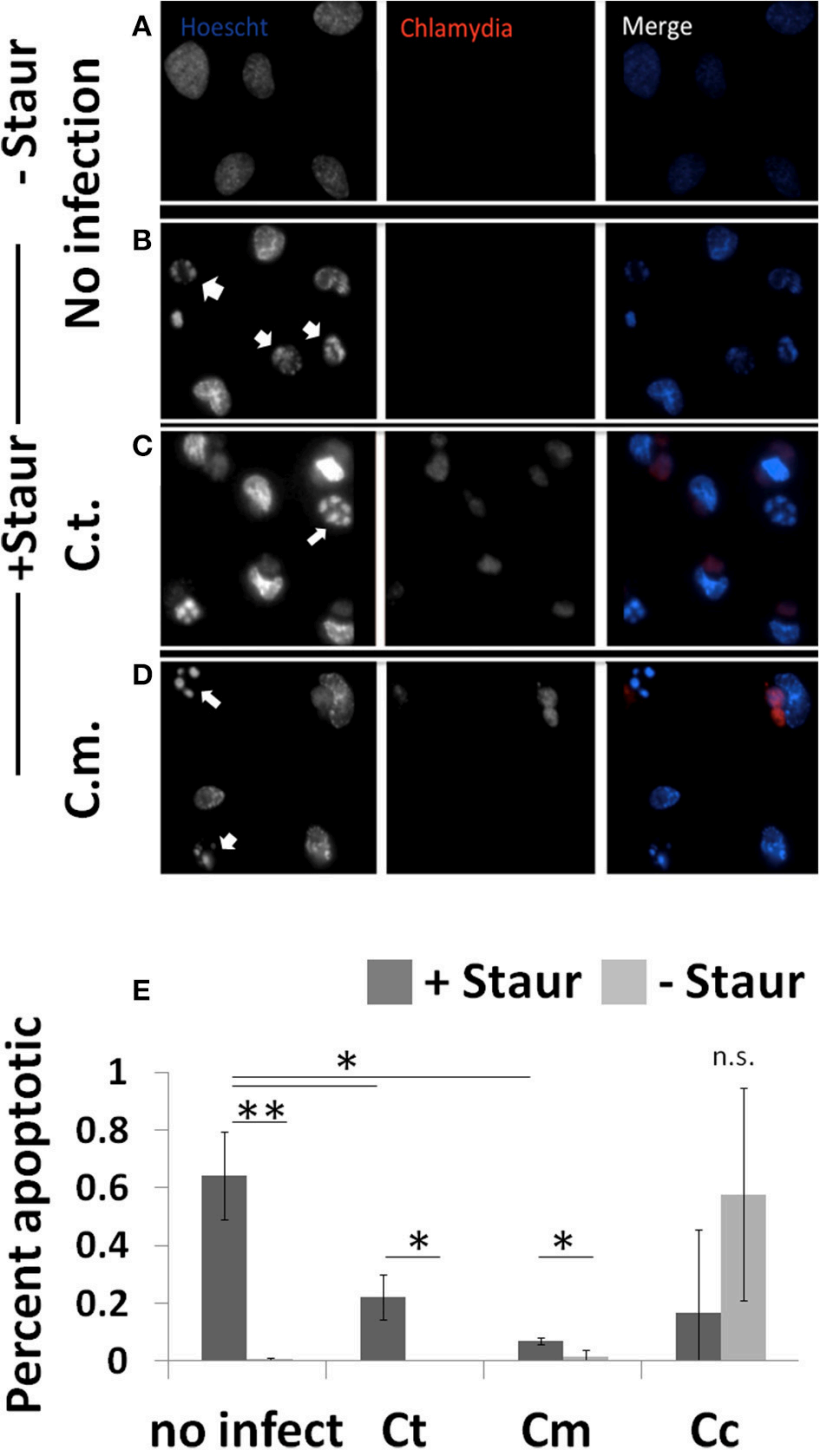
*****Chlamydiae*** inhibit nuclear fragmentation induced by staurosporine—Cell lines were plated on coverslips (A,B) or infected with pathogens for 26 h (C,D)**. Staurosporine was added for 4 h **(B–D)** before cells were fixed and stained to visualize *Chlamydiae* (αHSP-60) and DNA (Hoechst). White arrows indicate apoptotic cells. Percent apoptotic in each of the treatment conditions is quantified **(E)**. Ct-*C. trachomatis*, Cc-*C.caviae* Cm-*C.muridarum*. ^**^p < 0.01, ^*^p < 0.05 (representative of four independent trials).

To determine the extent to which *Chlamydiae* infection could impede nuclear fragmentation during apoptosis, cells were co-stained to identify both *Chlamydiae* inclusions and nuclear content (Figure 3). As shown in Figure 3B, when guinea pig cells are treated with staurosporine nuclear condensation occurs based on the observed increase in nuclear stain fluorescence and fragmentation of the nuclear material (*p* < 0.01). However, when these cells are infected with *Chlamydiae* the DNA fragmentation is inhibited (Figures 3C–E, *p* < 0.05). Cc infected cells were sparsely found on the slides due to apoptotic destruction of the cell prior to labeling leading to large variability and inability to image clusters of cells (Figure 3, Supplemental Figure 2C and data not shown). Collectively, these data indicate that *Chlamydiae* are able to inhibit the apoptosis of the host cell and that the level of success for the pathogen is dependent upon the species of host cell infected.

### *Chlamydiae* upregulate anti-apoptotic genes unique to each host-pathogen combination

We have established that *Chlamydiae* are capable of inhibiting apoptosis across multiple host species boundaries. However, the importance of host derived anti-apoptotic gene regulation during *Chlamydiae* infection has led to conflicting reports in the literature. Rajalingam et al. indicate that IAP complexes are highly upregulated in human cells following infection with Ct and these complexes are necessary for infection. Whereas Ying et al. report that Ct infection of mouse cells deficient in these IAP genes are still fully capable of inhibiting cellular apoptosis (Rajalingam et al., 2006; Ying et al., 2008). To rectify these incongruous results, we examined the regulation of anti-apoptotic transcripts in each of our host species cell lines when infected by *Chlamydiae*. Based on our earlier finding that the absence of CHX improves bacterial growth in certain cell lines, we decided to examine transcript levels in both the presence and absence of CHX.

**Figure 4:**
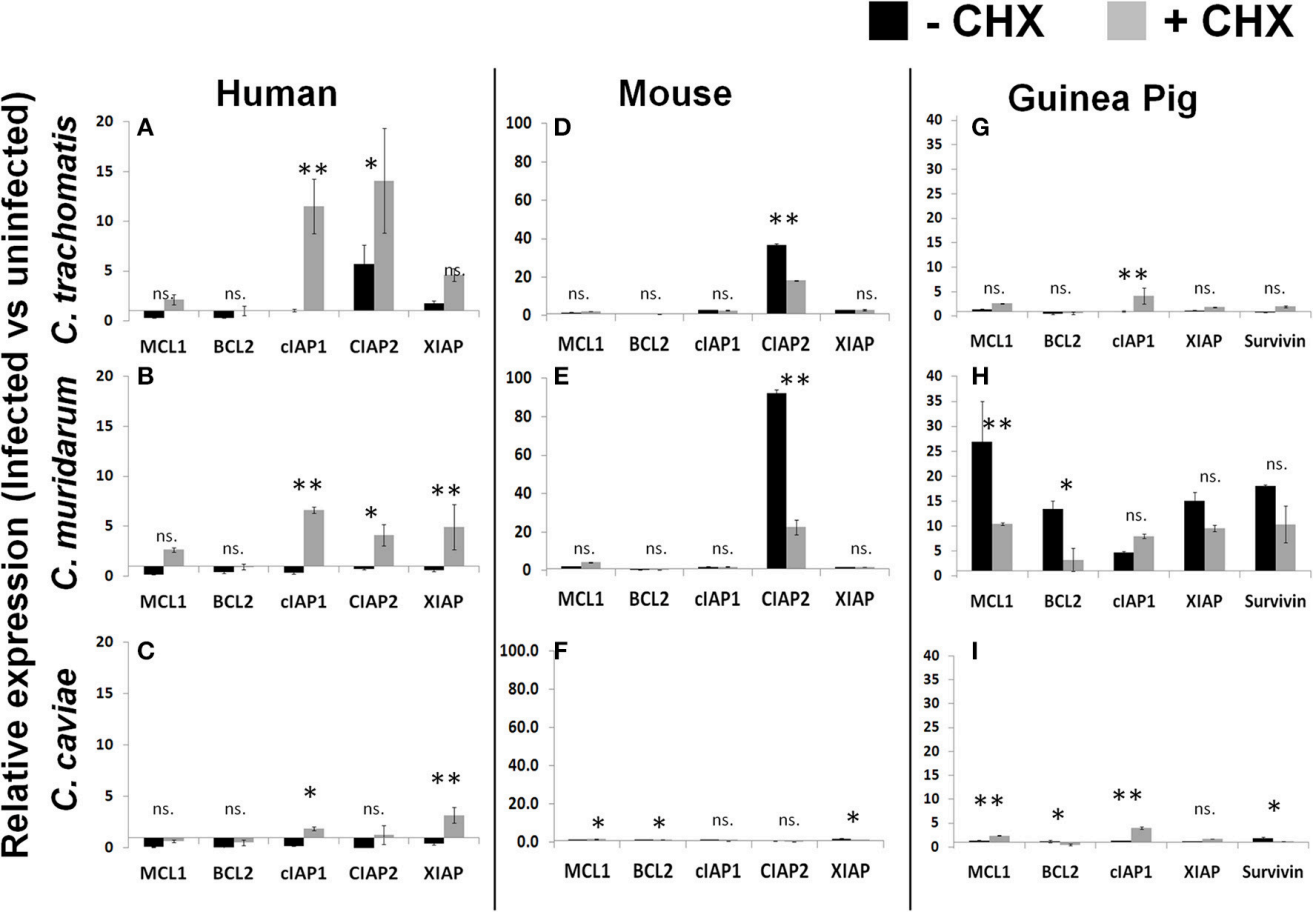
**Infection induced transcription of anti-apoptotic factors in the presence or absence of host translation**. Cell lines were plated in six well plates and infected for 30 h prior to lysis and RNA extraction. Relative expression was normalized to host GAPDH and then the ratio of infected vs. uninfected gene expression was determined in human cells **(A–C)**, mouse cells **(D–F)** and guinea pig cells **(G–I)**. ^**^p < 0.01, ^*^p < 0.05 (representative of three independent trials).

As was reported by Rajalingam, we identified an upregulation of IAP genes following infection of human cells with the Ct pathogen in both the presence and absence of CHX (Figure 4A). Cm and Cc infections of human cells also follow a very similar pattern of IAP regulation except these results are dependent on CHX treatment (Figures 4B,C). In contrast to human cells, infection of mouse cells with Ct or Cm were highly specific for cIAP2 upregulation and this regulation was significantly higher in cells which were actively able to translate host proteins (Figures 4D,E). Cc infection of mouse or human cells exhbited a marginal impact on the levels of antiapoptotic transcripts (Figure 4F). Thus, the specific antiapoptotic gene transcripts (and their regulation) are completely host species dependent and are, in some cases, unique to the infecting pathogen.

As described in Figure 1, Cm infection of guinea pig cells was significantly improved by the absence of CHX. When we examined the anti-apoptotic transcript expression level in these cells we found that Cm infection casued a very prominent upregulation, compared to Ct and Cc infection (Figures 4G–I). In particular, Mcl1 was very highly upregulated in guinea pig cells following Cm infection. Taken together, these data indicate that each pathogen species has a unique ability to impact the host transcription of anti-apoptotic genes. The quantity and diversity of this regulation was pathogen-species specific and the pattern of transcript expression was unique to each host species cell line.

## Discussion

*Chlamydiae* have been co-evolving with their hosts for the last several hundred million years (Nunes and Gomes, 2014). Throughout this time the competition between pathogen and host has led to co-adaptation as the pathogen struggles to maintain its intracellular replicative niche (Nunes et al., 2008). *Chlamydiae* can be distinguished based on their specific amino acid requirments with a level of specificity down to identification of *C. trachomatis* biovars requirmeent for tryptophan(trachoma) and methionine(LGV) supplementation of culture media (Allan and Pearce, 1983a). The mechanisms for aquiring amino acids can also be very different amongst *Chlamydiae*. In a direct comparison to *C. pneumoniae*, Ct L2 was shown to be better at accessing host amino acids in the presence of a lysosomal inhibitor (Ouellette et al., 2011). The authors conclude that *C. pneumoniae* and Ct L2 have inherent differences in their mechanisms of amino acid acquisition. The addition of CHX provides intracellular pathogens with reduced host competition for nutrients and suppresses any de-novo protein production the host may undertake in order to rid itself of the infection (Ripa and Mårdh, 1977; Benes and McCormack, 1982; Sabet et al., 1984; Harper et al., 2000). Under these optimal conditions, in the absence of competition, *Chlamydiae* can readily infect across host species boundaries. We asked if this “advantage” was absolutely necessary for highly host adapted pathogens to compete with the host for nutrients and, perhaps, the pathogen could utilize the host's own protein production to its advantage. To test for different levels of human adaptation, we compared two Ct pathogens which infect at the same site but have markedly different infectious phenotypes. The invasive Ct LGV strains exhibit rapid growth kinetics and capacity to infect systemically, as compared to the genital (or ocular) strains (Miyairi et al., 2006). Based on these data, we hypothesized that the LGV strains would be better at competing with the host for nutrients and would not require CHX treatment for optimal growth. Our data support this hypothesis since the provision of “free” amino acids following CHX treatment exhibited a marginal impact on LGV growth in HeLa cells as compared to the Ct-D strain (Supplemental Figure 3). These data support a similar finding when Ct-L2 was compared to Ct-E growth in conditions ± CHX (Harper et al., 2000). Extending this study to *Chlamydiae* infection across multiple host species of cells, we found that only the Cm infection of guinea pig cells demonstrated a unique species specific and significant growth advantage from ongoing host cell translation, compared to Ct and Cc infection (Figure 1A). In addition, Cm infection exhibits some of the fastest growth kinetics and largest replicative burst across multiple host species cell lines (Supplemental Figure 1). From this we infer that Cm, despite being considered a mouse specific *Chlamydia* pathogen, is actually a generalist due to its highly permissive growth characteristics, with or without CHX treatment. In contrast, Cc infections did particularly poorly across multiple hosts indicating that it is the least well-adapted to deal with the unique biology of these divergent host species.

One main host mechanism to deny the pathogen further growth potential is the organized cellular death pathway of apoptosis. As such, the *Chlamydiae* pathogens have evolved mechanisms to subvert this pathway and it has been the topic of significant investigation for several years (Byrne and Ojcius, 2004). A thorough investigation of multiple *Chlamydiae* species had previously identified a common capacity to inhibit apoptosis in diverse tissue types of human cell lines (Greene et al., 2003). We have extended these findings and demonstrate that *Chlamydiae* can inhibit apoptosis across multiple host species boundaries. We used activation of caspase three, the terminal caspase in the proteolytic cascade, and nuclear fragmentation as markers of apoptosis (Thornberry and Lazebnik, 1998). As demonstrated in Figure 2, we corroborate those earlier reports in that almost all *Chlamydiae* species are able to inhibit host cell apoptosis. Intriguingly, Cc, the pathogen isolated from guinea pigs, was the least capable to grow within guinea pig cells. Based on light microscopy analysis, Cc infection of guinea pig cells lead to significant membrane blebbing and apoptotic phenotypes (Supplemental Figure 2). This led to particular problems in acquiring Caspase 3 activity and nuclear condensation as the cells were easily lost with gentle washing. The variability of Cc infection in Figure 3E is due to this high rate of cell loss due to combined infection and staurosporine induced apoptosis. In most situations, Cc was the least capable of down regulating the host Caspase 3 activity as compared to Ct or Cm infection within each cell line (Figure 2).

The ability of *Chlamydiae* to subvert host cell apoptotic responses could occur through two potential mechanisms, modulation of intrinsic host cell anti-apoptotic responses or through a direct impact on host cell biology via a type three secreted virulence factor. The latter option has been well studied in that *Chlamydia* post-translationally impact BH3 domain pro-apoptotic proteins even in the presence of CHX (Fan et al., 1998; Fischer et al., 2004; Pirbhai et al., 2006). However, *Chlamydia* infection can also cause significant changes in host cell transcription (Hess et al., 2001). These changes to host cell biology could be induced through cellular recognition of *Chlamydia* via pattern recognition receptors (such as TLR2) or Raf/MEK signaling pathways (Su et al., 2003; O'Connell et al., 2006). *C. trachomatis* has developed measures to manipulate these signaling pathways, such as modulating the host ubiquitination system necessary for NF-kB signaling (Misaghi et al., 2006). NF-kB can upregulate proteins which inhibit apoptosis (IAPs) and are critical for cell survival following Ct infection (Rajalingam et al., 2006). RNAi inhibition of these IAPs lead to a less permissive cellular niche and limited replication of *Chlamydia*. Additionally, signaling through the Raf/MEK pathway leads to an upregulation of anti-apoptotic protein Mcl1 and Bag-1 (Rajalingam et al., 2008; Kun et al., 2013). When Raf/MEK pathways were inhibited, *Chlamydia* infected cells were highly susceptible to apoptosis induction. As an alternative to manipulating cell signaling pathways, *Chlamydiae* could use a type three secretion system (T3SS) to directly inject the host cytosol with a bacterial protein which homes to the eukaryotic nucleus and modifies host cell transcription. Although its function is unknown, CT311 has recently been demonstrated to contain a eukaryotic nuclear localization signal (Lei et al., 2013). It is clear that infection with *Chlamydiae* induces significant anti-apoptotic gene transcriptional changes in a host cell which can directly impact the viability of the host cell and its resistance to apoptosis induction.

The importance of anti-apoptotic protein production during *Chlamydia* infection has recently been called into question. Studies with cell lines derived from mice deficient for these IAPs indicate that the proteins are dispensable for the anti-apoptotic effect (Ying et al., 2008). Discrepancies in host cell species (human/mouse) and *Chlamydia* type were acknowledged by both reports as confounding factors (Rajalingam et al., 2008; Ying et al., 2008). To address the host species component of these incongruous results, we examined the regulation of anti-apoptotic transcripts in various host species cell lines across multiple *Chlamydiae* pathogens. We identified that a single host could respond differently to each *Chlamydiae* pathogen (Figure 4). Differences in gene expression between hosts could be due to host species differences or cell type differences. Therefore, inter-host species comparisons are not a focus of this study. In some cases the upregulation of anti-apoptotic protein transcripts required ongoing host translation, indicating a potential positive feedback loop induced by infection. Our transcriptional profile data does support that conclusion in some cases where, in the absence of CHX, anti-apoptotic protein transcripts are significantly augmented (Figures 4D–H). We would surmise that despite the similar outcome of blocked apoptosis, each pathogen species reaches that goal via a potentially different mechanism. Intriguingly, the mouse cell line predominantly upregulated a single anti-apoptotic transcript, cIAP2, which was shown to be dispensable (Ying et al., 2008). This would indicate that, in mice, an alternative method for apoptosis inhibition could be employed.

Rather than rely solely on host cell signaling pathways for the anti-apoptotic effect, evidence indicates that *Chlamydiae* utilize a T3SS to inject bacterial proteins which directly impact host cell biology. Early evidence indicated that *Chlamydia* could induce proteolytic cleavage of pro-apoptotic BH3-only proteins (Fischer et al., 2004; Zhong et al., 2006). This proteolysis was attributed to the *Chlamydia* protease-like activity factor (CPAF) (Pirbhai et al., 2006; Paschen et al., 2008) However, the role of CPAF in this mechanism has been called into doubt following the development of *Chlamydia* genetically deficient for protease activity (Jorgensen et al., 2011; Snavely et al., 2014). Moreover, this BH3 protein cleavage mechanism was not reproducible (Rajalingam et al., 2008). The study of pro-apoptotic BH3-only protein cleavage would also benefit from an evolutionary approach to examining host-pathogen interactions and address the discrepancies in these reported systems.

Pathogens co-adapt with their hosts throughout evolution to maintain their capacity for growth and dissemination. Similar evolutionary medicine studies examining host specificity in *Salmonella* infection have helped to identify key virulence factors in host specificity (Bueno et al., 2008; Bäumler and Fang, 2013). For *Chlamydiae*, the relatively small genomes add significant evolutionary pressure due to gene loss, alternatively termed the “use it or lose it” impact of adaptation (Nunes and Gomes, 2014). These host species-specific adaptations could impair the pathogen's ability to leap across species boundaries and emerge as an infectious disease in a new host. However, data indicate one such emergence has occurred fairly recently in humans with the zoonotic acquisition *C. pneumoniae*, demonstrating *Chlamydiae* are a good model for the study of emerging infectious disease (Myers et al., 2009). The evolutionary medicine approach will be highly informative to understanding the barriers for host species-to-species pathogen transmission. The experiments in this report begin to elucidate the potential mechanisms whereby *Chlamydiae* are able to cross the species boundary and provide a greater understanding of the barriers to emerging infectious disease.

## Author contributions

JM, EN, CF, DG designed, performed, and analyzed the experiments for this manuscript. JM, EN, DG drafted and CF, DG revised the manuscript. All authors agree to be accountable for all aspects of the work herein.

### Conflict of interest statement

The authors declare that the research was conducted in the absence of any commercial or financial relationships that could be construed as a potential conflict of interest.
