# Supplementary figures and images for: Chlamydia Infection Across Host Species Boundaries Promotes Distinct Sets of Transcribed Anti-Apoptotic Factors
Source: Front Cell Infect Microbiol. 2015 Dec 23;5:96. doi: 10.3389/fcimb.2015.00096 (PMC4688367; doi:10.3389/fcimb.2015.00096)

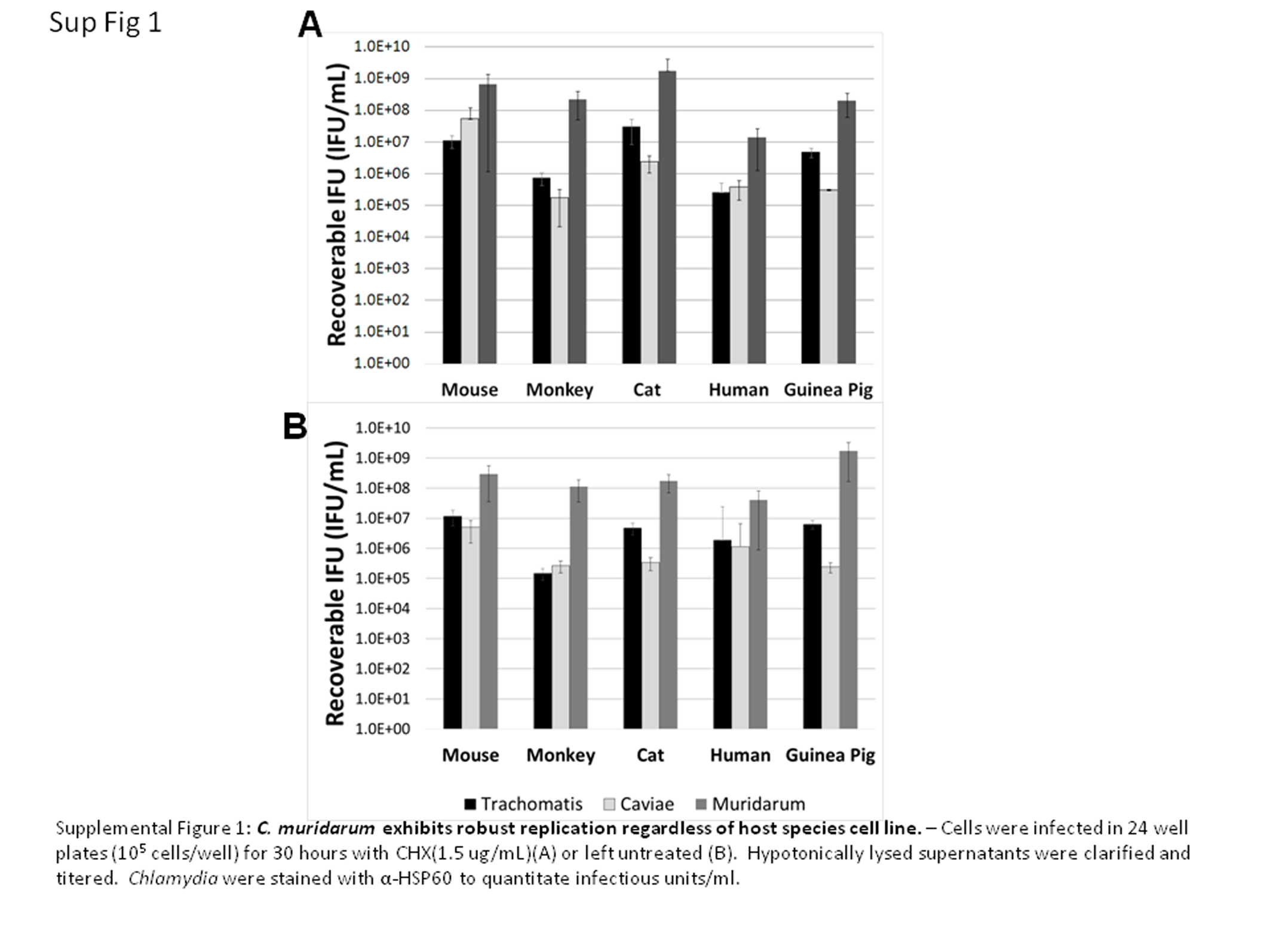

Supplement: Supplementary file 1 [file Image1.TIF]

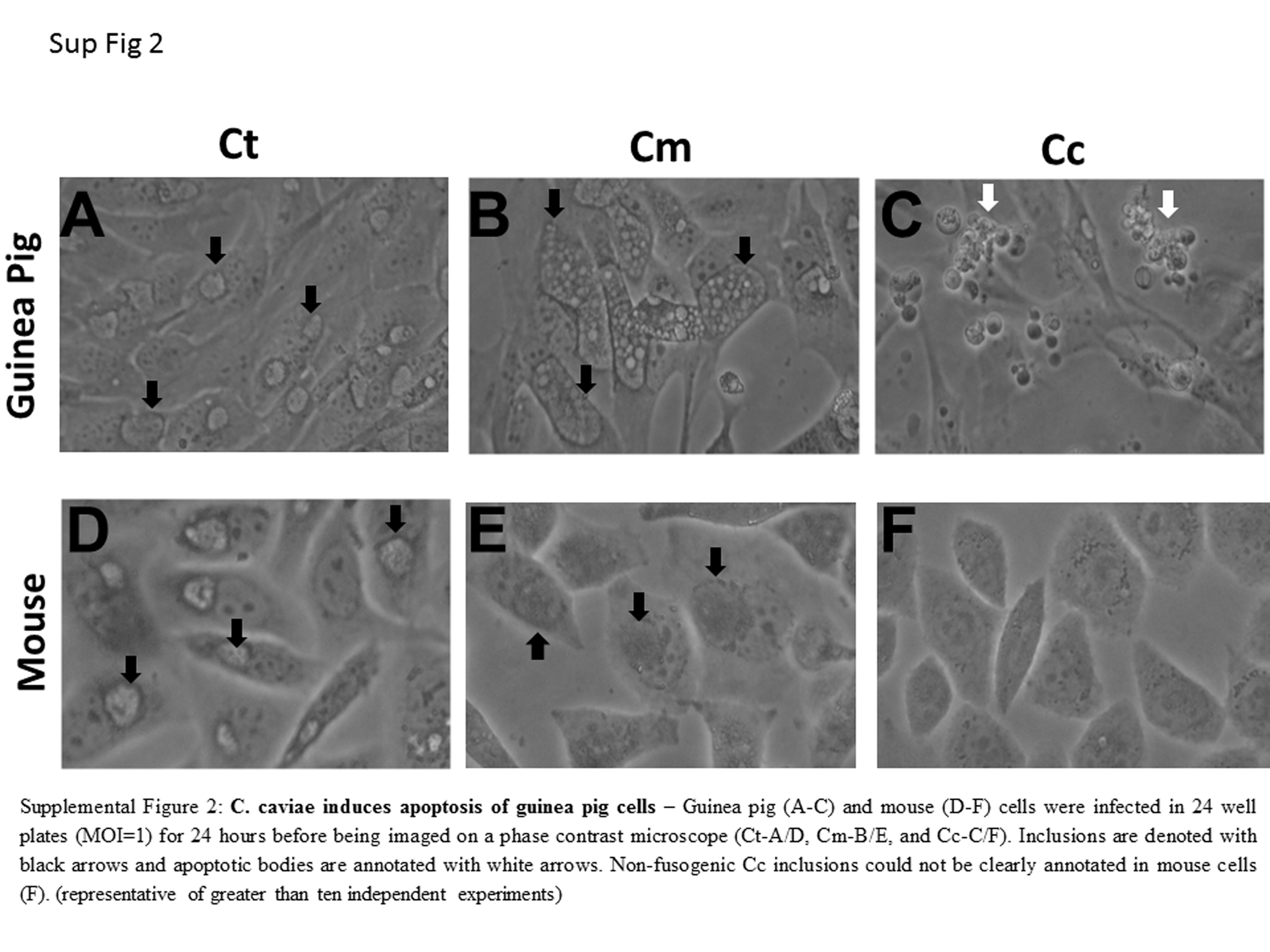

Supplement: Supplementary file 2 [file Image2.TIF]

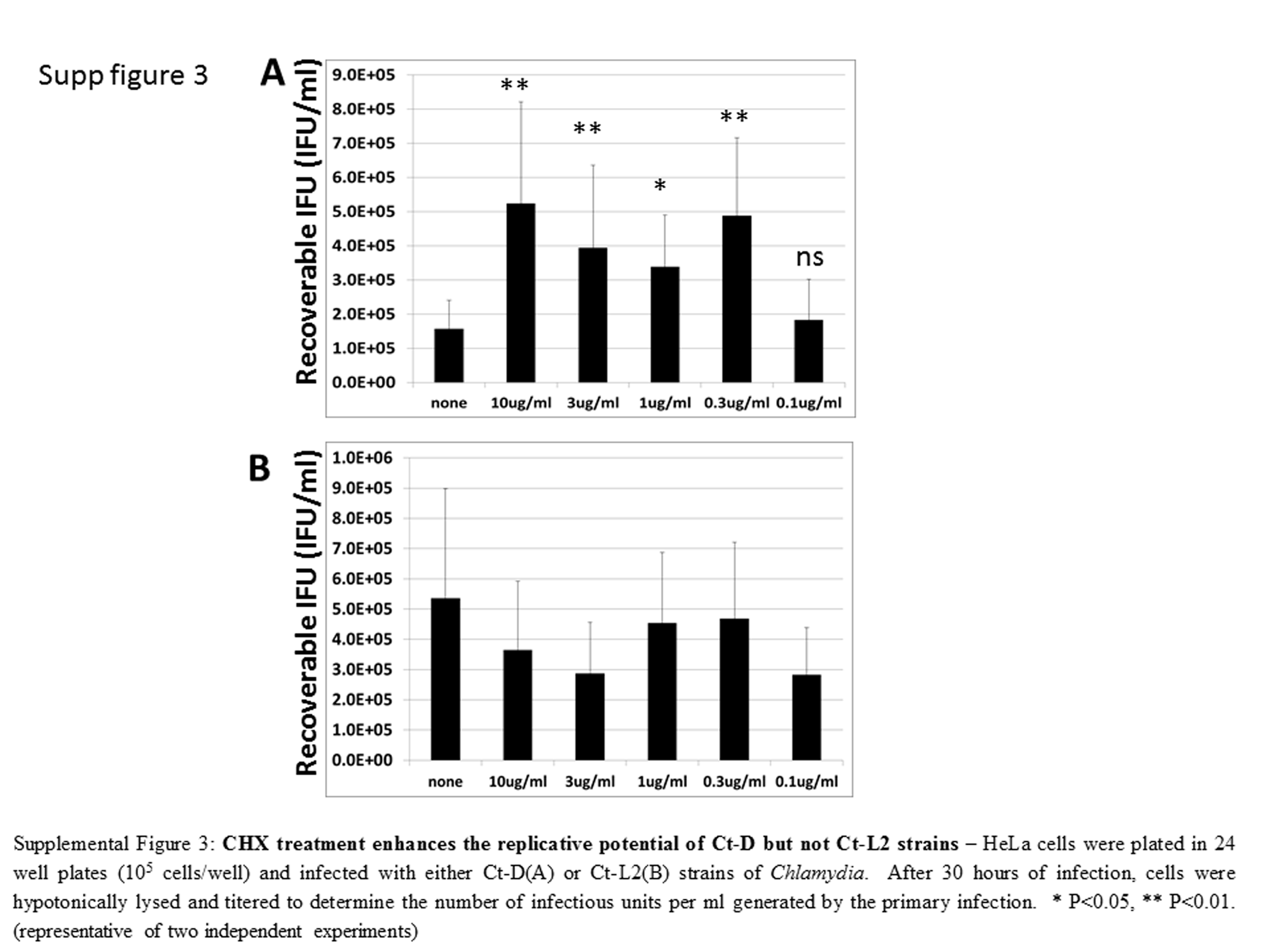

Supplement: Supplementary file 3 [file Image3.TIF]
